# ‘DURVIT’: a phase-I trial of single low-dose durvalumab (Medi4736) IntraTumourally injected in cervical cancer: safety, toxicity and effect on the primary tumour- and lymph node microenvironment

**DOI:** 10.1186/s12885-018-4764-0

**Published:** 2018-09-12

**Authors:** J. Rotman, C. H. Mom, E. S. Jordanova, T. D. de Gruijl, G. G. Kenter

**Affiliations:** 1 Amsterdam UMC, Vrije Universiteit Amsterdam, Department of Obstetrics and Gynaecology, Cancer Center Amsterdam, Center for Gynaecologic Oncology Amsterdam (CGOA), CCA 2.48, De Boelelaan 1117, 1081 HV Amsterdam, the Netherlands; 2 Amsterdam UMC, Univ(ersity) of Amsterdam, Department of Obstetrics and Gynaecology, Center for Gynaecologic Oncology Amsterdam (CGOA), H4-222, Postbus 22660, 1100 DD Amsterdam, the Netherlands; 3 Amsterdam UMC, Vrije Universiteit Amsterdam, Department of Medical Oncology, Cancer Center Amsterdam, CCA 2.44, De Boelelaan 1117, 1081 HV Amsterdam, the Netherlands; 4 The Netherlands Cancer Institute - Antoni van Leeuwenhoek Hospital, Department of Gynaecology, Center for Gynaecologic Oncology Amsterdam (CGOA), Plesmanlaan 121, 1066 CX Amsterdam, the Netherlands

**Keywords:** Immunotherapy, Cervical cancer, Durvalumab, Programmed cell death ligand 1, Checkpoint inhibitor, Local therapy, Intratumoural

## Abstract

**Background:**

Treatment with programmed cell death receptor (PD-1) and programmed cell death ligand 1 (PD-L1) inhibitors is a promising strategy to lift tumour-induced immune response suppression. However, the current systemic treatment often causes autoimmune side effects. In more than 50% of squamous cell cervical cancer, PD-L1 expression is detected. Moreover, we observed high and interrelated rates of PD-L1 positive macrophages and regulatory T cells in metastatic lymph nodes of cervical cancer patients. As cervical cancer in general initially metastasizes to regional lymph nodes, local administration of durvalumab (a PD-L1 checkpoint inhibitor) at an early stage will deliver these antibodies exactly where they are needed, facilitating immune protection. This may result in a clinical benefit while reducing undesirable side effects.

**Methods:**

DURVIT is a non-randomized, single-arm, open-label, phase I study. Three escalating dose levels of intratumourally (i.t.) injected durvalumab will be tested, i.e. 5, 10 and 20 mg (three patients per dose level, with an additional three at the highest tolerated dose). The primary endpoint of this phase-I study is safety. Immune monitoring will consist of flow cytometric, immunohistochemical and functional T cell reactivity testing. The first patient has been included in this trial in November 2017.

**Discussion:**

Evidence of safety and biological efficacy of this locally administered checkpoint blockade may expand adjuvant therapy options for cervical cancer patients. Early metastatic spread of cervical cancer cells may thus be controlled in the draining lymph node basin, and beyond, and hopefully delay or even prevent the onset of disease recurrence.

**Trial registration:**

NTR6119, 1-nov-2016.

**Electronic supplementary material:**

The online version of this article (10.1186/s12885-018-4764-0) contains supplementary material, which is available to authorized users.

## Background

Cervical cancer is the fourth most common cancer in women worldwide and is caused by a persistent infection with high-risk human papilloma virus (HPV) types [1, 2]. The highest incidence of cervical cancer lies between 35 and 45 years of age [3]. Although vaccines to prevent cervical cancer are widely implemented, advanced stage cervical cancer is still an important cause of mortality among women worldwide [4].

The most important prognostic factor in early stage cervical cancer is the presence of metastatic tumour cells in the pelvic lymph nodes [5]. After radical hysterectomy and pelvic lymphadenectomy, women with early stage cervical cancer with negative lymph nodes have a 5-year survival rate of 80–90%, compared to a 5-year survival of 60–65% for patients with one lymph node metastasis [6, 7]. Adjuvant treatment in patients with lymph node metastasis and/or other risk factors is (chemo)radiation [8, 9]. However, adjuvant chemoradiation is associated with increased morbidity (with reported symptoms such as nausea, pain, vaginal tightness and urinary complaints) and impaired quality of life [10]. Of note, adjuvant (chemo)radiation in cervical cancer may also result in ovarian failure, and most patients diagnosed with cervical cancer are relatively young [11].

To improve the prognosis and quality of life of cervical cancer patients, novel adjuvant treatments are urgently needed. A highly promising area of research focuses on lifting tumour-induced immune suppression. Cancer cells employ various mechanisms to evade immune-mediated surveillance and elimination, which allows them to develop and spread unchecked. One of these strategies comprises upregulation of proteins on the cell surface that deliver inhibitory signals to cytotoxic T cells, the so-called immune checkpoints. Programmed cell death ligand 1 (PD-L1) is an example of such an immune checkpoint, and is upregulated in a broad range of cancers, including lung [12], renal cell [13–15], pancreatic [16–18], ovarian cancer [19] and hematologic malignancies [20, 21].

Several studies have reported on the upregulation of PD-L1 and/or PD-1 in cervical carcinoma and surrounding inflammatory cells [22–25]. Recently, we performed a retrospective study on primary tumours (*n* = 205) and paired metastatic lymph nodes (*n* = 127) from cervical cancer patients and showed PD-L1 expression by primary tumour cells as well as by tumour infiltrating and stromal CD163+ positive M2 macrophages [26]. In 54% of all squamous cell primary tumours (SCC) and in 14% of all adenocarcinomas (AC) PD-L1 positivity was observed in > 5% of the tumour cells. PD-L1 expression in tumour margins (i.e. at the tumour/stroma interphase) in SCC was related to favourable survival and most likely induced by IFNγ released by adjacent activated T cells. In SCC, diffuse PD-L1 expression was associated with poor prognosis as was the presence of PD-L1 positive macrophages in AC. Furthermore, we reported on the high and interrelated rates of PD-L1 positive myeloid cells and regulatory T cells (Tregs) in metastatic lymph nodes in patients with cervical cancer [27]. In a comparative study of the immune status of all dissected cervical tumour-draining lymph nodes in five patients, we described that immunosuppression (identified as low CD8+ T cell/ FoxP3+ Treg ratios) may precede actual metastasis, creating niches in the tumour-draining lymphatic catchment area [28]. These results led to the hypothesis that tumour-associated PD-L1 positive macrophages expand Tregs which subsequently migrate to down-stream lymph nodes to create immune suppressed metastatic niches [29].

These studies support the clinical exploration of immunotherapies aimed at counteracting the immunosuppressive microenvironment in the primary tumour and the tumour-draining lymph nodes by PD-1/PD-L1 checkpoint blockade. By facilitating a robust antitumour T cell response, immune therapy can break the cycle of immune suppression and metastatic spread.

Durvalumab is a human monoclonal antibody (mAb) of the immunoglobulin G1 kappa (IgG1κ) subclass that blocks binding of PD-L1 to PD-1 and CD80 (B7–1). To date, results of several trials with systemically administered durvalumab in patients with advanced or metastatic cancer show promising antitumour activity with durable responses [30]. Durvalumab was recently approved by the U.S. Food and Drug Administration for patients with locally advanced or metastatic urothelial carcinoma who have disease progression during or following platinum-containing chemotherapy [31].

The systemic treatment with PD-1 and PD-L1 inhibitors can cause severe autoimmune side effects [32]. In the current study durvalumab is administered locally, i.e. in the cervix. As cervical cancer initially metastasizes through regional lymph nodes, we believe that local administration of durvalumab at an early stage will deliver these antibodies exactly where they are needed. Our hypothesis is that local conditioning of the tumour and tumour draining lymph nodes (TDLN) in the neo-adjuvant setting will lead to both loco-regional and systemic immune activation.In this way, undesirable systemic side effects may be avoided. Additional interest in local administration of checkpoint inhibitors is raised by the fact that the locally administered medication is expected to be (systemically) effective at a lower dose, leading to a desirable decline in the expenses involved.

## Methods/design

### Study design

**Fig. 1:**
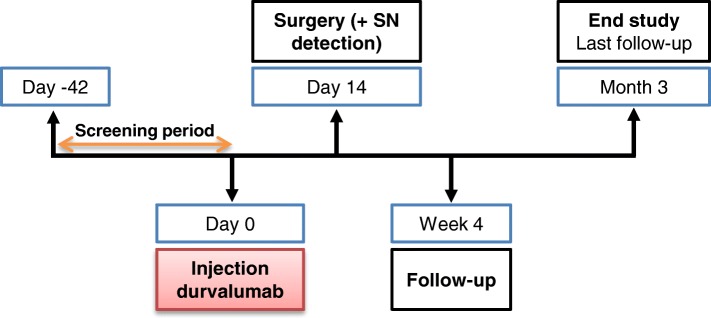
DURVIT study timeline. SN = sentinel lymph node

**Fig. 2:**
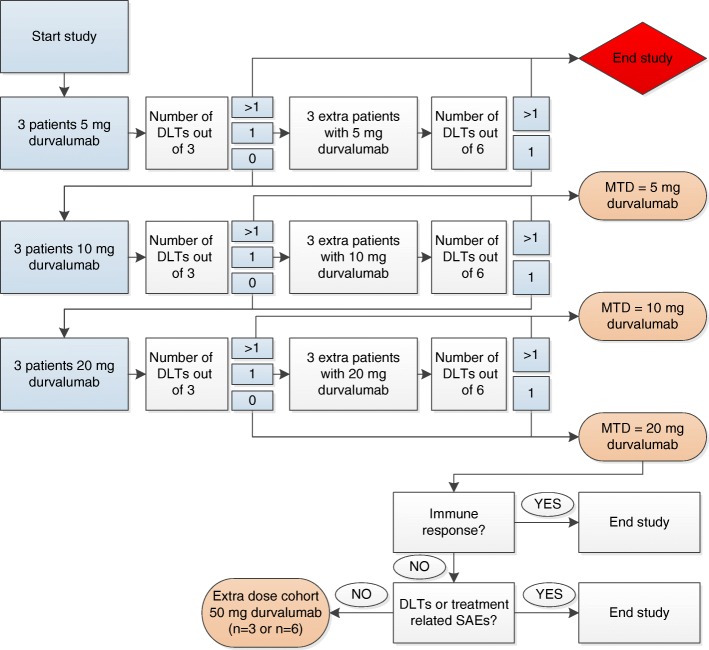
DURVIT study flowchart. DLT = dose limiting toxicity, SAE = serious adverse event, MTD = maximum tolerated dose

‘DURVIT’ is a non-randomized, single-arm, open-label, phase I study. Patients with cervical cancer who are scheduled for (radical) hysterectomy with lymph node dissection will be enrolled at the Amsterdam UMC (formerly: Academic Medical Center (AMC), Amsterdam). Two weeks before the patient is scheduled for surgical treatment, durvalumab (AstraZenecaBV) will be injected locally into the cervix (Fig. 1). Three doses of durvalumab will be tested in a 3 + 3 dose escalation design: 5, 10 and 20 mg intratumourally (i.t.) (Fig. 2). If no dose limiting toxicities (DLTs) or treatment related serious adverse events (SAEs) are observed in the 3 different dose cohorts (5, 10, 20 mg) and no clear (systemic) immunological responses are detected based on T cell levels and FACS immunomonitoring, we will add an extra dose cohort of 3 patients treated with 50 mg durvalumab i.t. based on the same criteria. The Common Terminology Criteria for Adverse Events (CTCAE) v4.03 will be used for the assessment of adverse events.

Pre-treatment archival formalin-fixed, paraffin-embedded (FFPE) tissue samples will be requested from centres where patients were diagnosed or had their primary treatment. Post-surgery tumour and lymph node material will be fixed and stored. To have a complete clinical characterization of the patients included in this study, we will perform the standard diagnostic HPV typing for our institution: the HPV Risk Assay (Self-Screen) [33]. Blood samples will be taken once during the screening period, at day 0 (prior to durvalumab administration, i.e. at baseline), at day 14 (at the time of surgery), after 4 weeks, and at 3 months after administration of durvalumab. Post-surgery biopsies of the removed tumour and sentinel and non-sentinel TDLN samples will be collected and processed as described previously [27].

The trial has been approved by the Institutional Review Board of the Academic Medical Center (AMC) and sponsored by the AMC, with funding from Stichting Vrije Universiteit Medical Center - Cancer Center Amsterdam (VUmc-CCA) and Astra Zeneca for the immunological tests (requested). Electronic data is submitted by the study staff via the online database CastorEDC. The study will be monitored by the Clinical Research Unit of the AMC. The first patient has been included in this trial in November 2017.

### Participants

The inclusion and exclusion criteria for the DURVIT-study are listed in Table 1.

**Table 1:** DURVIT Inclusion/Exclusion criteria

Inclusion criteria	Exclusion criteria
1. Age > 18 years at time of study entry 2. Willing and able to undergo the planned study procedures 3. World Health Organization (WHO) performance status of 0 or 1 4. Written informed consent 5. Histologically confirmed cervical cancer of all histological types 6. Scheduled to undergo (radical) hysterectomy with lymphadenectomy 7. No indication of an active infectious disease: HIV, HCV and HBV negative 8. No history of autoimmune disease or systematic underlying disease which might affect immunocompetence 9. Adequate bone marrow function 10. Subjects must either be of non-reproductive potential or must have a negative urine pregnancy test before study entry 11. Ability of subject to understand Dutch language	1. Prior treatment with immunotherapy including therapeutic vaccines 2. Involvement in the planning and/or conduct of the study 3. Participation in a study with another investigational drug within 30 days prior to enrolment in this study 4. Major surgery within 28 days before inclusion (conization or biopsy is not major surgery) 5. Severe cardiac, respiratory, or metabolic disease 6. Use of oral anticoagulant drugs (except ascal) 7. Severe infections requiring antibiotics 8. Lactation or pregnancy 9. Current or prior use of immunosuppressive medication within 28 days before the first dose of durvalumab, with the exceptions of intranasal and inhaled corticosteroids or systemic corticosteroids at physiological doses, which are not to exceed 10 mg/day of prednisone, or an equivalent corticosteroid 10. Any prior Grade ≥ 3 immune-related adverse event (irAE) while receiving any previous therapy, or any unresolved irAE >Grade 1 11. Active or prior documented autoimmune disease within the past 2 years 12. Active or prior documented inflammatory bowel disease 13. History of primary immunodeficiency/allogeneic organ transplant/previous clinical diagnosis of tuberculosis/uncontrolled intercurrent illness 14. Receipt of live attenuated vaccination within 30 days prior to study entry or within 30 days of receiving durvalumab 15. Any condition that, in the opinion of the investigator, would interfere with evaluation of study treatment or interpretation of patient safety or study results

### Interventions

Dependent on the dose cohort, 5, 10 and 20 mg (and possibly 50 mg) of durvalumab, in a 4 ml dilution will be administered using a single syringe and a 27-gauge needle. The solution will be administered at room temperature and will be injected at 4 sites (1 mL/site) peri- and/or intratumourally, depending upon tumour location, visibility and size. The injection procedure is identical to the i.t. injections already performed in a standardized fashion for the sentinel lymph node procedure. The whole procedure will take approximately 15–30 min.

During surgery (day 14), patent blue will be injected intratumourally for identification of the sentinel lymph node. The detection of the sentinel node using a blue dye and/or radioactive tracer is a feasible technique in cervical cancer [34] and increasingly used in the treatment of cervical cancer patients.

### Outcome measurements

The primary outcome of this study is safety, by the evaluation of (serious) adverse events, in order to determine the maximum tolerated dose (MTD) durvalumab. Dose-limiting toxicities (DLTs) will be evaluated during the dose escalation phase of the trial. If ≥2 out of 3 patients or ≥ 2 out of 6 patients in the first dose cohort (5 mg durvalumab) experience a DLT, this study will be ended. A DLT will be defined as any grade 3 or higher toxicity that occurs during the DLT evaluation period. Toxicity that is clearly and directly related to the primary disease or to another etiology is excluded from this definition. Grading of DLTs will follow the guidelines provided in the Common Terminology Criteria for Adverse Events (CTCAE) version 4.03.

The following will be DLTs:

- Any grade 4 immune-related adverse event (irAE)
- Any ≥ grade 3 colitis
- Any grade 3 or 4 non-infectious pneumonitis irrespective of duration
- Any grade 2 pneumonitis that does not resolve to ≤ grade 1 within 3 days of the initiation of maximal supportive care
- Any grade 3 irAE, excluding colitis or pneumonitis, that does not downgrade to grade 2 within 3 days after onset of the event despite optimal medical management including systemic corticosteroids or does not downgrade to ≤ grade 1 or baseline within 14 days
- Liver transaminase elevation > 8 × upper limit of normal (ULN) or total bilirubin > 5 × ULN
- Any ≥ grade 3 non-irAE, except for the exclusions listed in Additional file 1

The period for evaluating DLTs will be from the time of administration of durvalumab until 3 months afterwards.

Secondary outcomes include the analysis of the microenvironment and immune status of the primary tumour and the draining lymph nodes, as well as the systemic antitumour immune response. Tumour and TDLN single-cell suspensions, as well as peripheral blood mononuclear cells (PBMC), will be analysed by multiparameter FACS panels for frequency and activation state of dendritic cell subsets, myeloid derived suppressor cells, macrophages, effector-T cells and Tregs. Advanced 35-parameter CYTOF analyses will also be performed to delineate known as well as novel immune subsets. In this way, the effects of the loco-regional treatment with durvalumab will be ascertained.

State-of-the-art 7-parameter fluorescence immunohistochemistry (IHC) panels will be used to analyse lineage and activation markers for the same subsets as for the aforementioned flow cytometry panels. The use of pre- and post-treatment FFPE material will allow the precise analysis of the density, compartmentalization, and (co-)localization of specific subsets. All IHC parameters will be determined using fully automated analyses.

As an indication of the induction of local and systemic T cell immunity, IFNγ elispot assays after in vitro stimulation will be performed on TDLN single-cells and PBMC to ascertain pre- and post-treatment frequencies of HPV-specific T cells (against long peptide pools derived from the immunodominant region of HPV-16 E6). We will also assess HPV16 T cell reactivity in PBMC and small tumour and TDLN samples using an ultra-sensitive technique based on DNA-barcoded MHC multimers with a PCR-based read-out (in collaboration with Dr. Sine Reker Hadrup, Technical University of Denmark). The multimers will be complexed to synthetic peptides (9–10 aa, 1210 peptides in total) spanning the whole sequence of E2, E6, and E7.

### Considerations for sample size

In the maximum tolerated dose (MTD) level we will treat 3 additional patients (*n* = 6 in total). This number is based on a power calculation (α = 0.05, power = 0.8) to enable detection of a 33% decrease, as compared to untreated tumours, in the expression of Treg frequencies in the primary tumour based on recent data (unpublished) showing 38.1% Tregs (SD of 10.5) of all CD4+ T cells present in primary cervical tumours (*n* = 5). If no DLTs or treatment related SAEs are observed in the 3 different dose cohorts (5, 10, 20 mg) and no clear (systemic) immunological responses are detected based on T cell levels and FACS immunomonitoring, we will add an extra dose cohort of 3 patients treated with 50 mg durvalumab i.t. based on the same criteria as stated in this protocol. Therefore the sample size is at minimum 3 patients and at maximum 24 patients.

### Statistical analysis

Tabular summaries will be presented by cohort. Categorical data will be summarized by the number and percentage of subjects in each category. Continuous variables will be summarized by descriptive statistics.

#### MTD evaluation

The MTD will be based on the occurrence of any DLTs. Any DLTs will be summarized or listed.

#### Analysis of safety endpoint(s)

Safety analyses will include AEs, SAEs, changes in laboratory findings, vital signs, performance status and physical examinations. The number of patients reporting (S)AEs will be summarized. The treatment related (S)AEs will be summarized as well. Adverse events will be graded according to the NCI CTCAE v4.03. Similarly, laboratory abnormalities will be graded according to the NCI CTCAE v4.03, if applicable.

#### Analysis of secondary endpoints (immune parameters)

Frequencies and activation status of the aforementioned immune cell subsets will be analyzed before and after treatment. We will use standard paired or unpaired parametric T or non-parametric Mann Whitney U tests for comparisons between groups and one-way repeated measures ANOVA for follow-up analyses over time

## Discussion

With a peak incidence between 35 and 45 years of age, patients diagnosed with cervical cancer are relatively young. In early stage cervical cancer, the percentage of relapses is 5% to 40% depending on lymph node metastasis and other risk factors [35]. The most common types of cervical cancer are squamous cell carcinoma (SCC) and adenocarcinoma (AC), which are known to be mostly HPV-16 and HPV-18 positive, respectively [36, 37]. We have previously reported on the expression of PD-L1 in SCC and AC [26]. Intratumoural injection of anti-PD-L1, in this case durvalumab, with the aim to specifically modulate the loco-regional environment is an innovative clinical approach for the treatment of cervical cancer. Evidence of safety and biological efficacy of this strategy will contribute to the design of novel adjuvant therapy options for cervical cancer patients. In this way early metastatic spread to the draining lymph node basin, and beyond, may be controlled and thereby the onset of disease recurrence may be delayed or even prevented.

Although blocking the PD-1/PD-L1 axis has been associated with improved survival in many cancer types, auto immune-related side effects are often reported (in up to 70% of patients) [38]. These findings are based on studies including patients with advanced stages of disease. In studies evaluating the safety of intravenous anti-PD-L1 in patients with different types of cancer, most reported side effects were fatigue, infusion reactions, rash, arthralgia, pruritus, diarrhoea and decreased appetite. Immune-related adverse events included rash, hypothyroidism and hepatitis [39, 40]. Local administration of low-dose checkpoint inhibitors may reduce these side effects.

Until now, there is only one study that has reported interim results on the effect of systemic anti-PD-1 or PD-L1 treatment in cervical cancer. Data show that pembrolizumab, an anti-PD-1 antibody, can have durable antitumour activity in patients with PD-L1-positive advanced cervical cancer [41]. The safety profile was consistent with that seen in other tumour types. Out of 24 treated patients there were 2 discontinuations due to grade 3 treatment-related AEs and no ≥ grade 4 treatment-related AEs were reported. Currently, several trials are in progress testing systemic anti-PD-1 or PD-L1 treatment in cervical cancer [42]. However, none of these trials concern the local administration of therapy.

We have recently obtained promising results in early-stage melanoma patients receiving a single low dose of the anti-CTLA4 checkpoint inhibitor tremelimumab. Low Treg frequencies were seen in the draining sentinel lymph node (compared to historic saline placebo controls) as well as post-treatment reduced systemic rates of activated Tregs in peripheral blood. Simultaneously, tumour-specific, NY-ESO-1 reactive effector-T cell frequencies were increased in the peripheral blood. Of note, apart from one mild case of vitiligo, no serious side effects were observed [van Pul et al., manuscript in preparation]. Furthermore, in 2016 Ray et al. reported that intratumourally injected IL-2 and ipilimumab (anti CTLA-4) in patients with non-resectable melanoma was well tolerated. Antitumour responses were detected in the injected lesions, as well as an abscopal effect was observed [43]. In general, anti-CTLA4 checkpoint inhibitors have more immune related adverse events than antibodies blocking PD-1 or PD-L1 [38].

Encouragingly, similar local immune potentiation of the primary melanoma excision site and the sentinel lymph node with the Toll-like receptor-9 ligand CpG-B in two randomized phase II trials of early-stage patients with melanoma led to activation of dendritic cell subsets. Tumour-specific T cell expansion at the injection site, in the draining sentinel lymph nodes and peripheral blood was detected [44, 45] Moreover, significantly increased recurrence-free survival rates were observed [46].

Since patients will be given a single and low dose of durvalumab, we do not expect any treatment related SAEs exceeding grade 3. We anticipate that the side effects of local administration of durvalumab may include:

- local inflammation reaction of the vagina, vulva and/or cervix with one or more of the following symptoms:

- ○ change in the volume, consistency, colour, or odour of vaginal discharge
- ○ vulvar or vaginal irritation, or burning sensation
- ○ pruritus
- ○ dysuria
- ○ genital edema

- hemorrhage or fistula due to tumour or tissue necrosis/degeneration.

## Conclusion

For the first time in cervical cancer, intratumoural administration of an immune checkpoint inhibitor will be investigated primarily for safety. We believe we will also be able as an exploratory objective, to unravel in a quantitative and qualitative manner the effect of the PD-L1 inhibitor durvalumab on the microenvironment in the primary tumour, the tumour draining lymph nodes and, importantly, on the systemic immune response. The proposed correlative immunoassays will shed light on mechanisms underlying the biological effects of PD-L1 blockade and may demonstrate its biological efficacy. These tests will aid in the selection of optimal dose and target population for subsequent studies, and facilitate a rational approach to the design of later phase 2 trials of this novel immunotherapy strategy.

## Additional file

Additional file 1: List of ≥ grade 3 non-irAE excluded for the definition of DLT. (DOCX 15 kb)

## Abbreviations

AC: Adenocarcinoma
AE: Adverse events
AMC: Academic Medical Center
CTCAE: The Common Terminology Criteria for Adverse Events
DLT: Dose limiting toxicity
FFPE: Formalin-fixed paraffin-embedded
HPV: Human papilloma virus
i.t.: Intratumoural
IgG1κ: Immunoglobulin G1 kappa
IHC: Immunohistochemistry
irAE: Immune-related adverse event
mAb: Monoclonal antibody
MTD: Maximum tolerated dose
PBMC: Peripheral blood mononuclear cells
PD-L1: Programmed cell death ligand 1
SAE: Serious adverse event
SCC: Squamous cell carcinoma
TDLN: tumour draining lymph node
Treg: Regulatory T cell
ULN: Upper limit of normal
