# Supplementary material for: ‘DURVIT’: a phase-I trial of single low-dose durvalumab (Medi4736) IntraTumourally injected in cervical cancer: safety, toxicity and effect on the primary tumour- and lymph node microenvironment
Source: BMC Cancer. 2018 Sep 12;18:888. doi: 10.1186/s12885-018-4764-0 (PMC6134598; doi:10.1186/s12885-018-4764-0)
Supplement: Supplementary file 1 — List of ≥ grade 3 non-irAE excluded for the definition of DLT. (DOCX 15 kb) [file 12885_2018_4764_MOESM1_ESM.docx]

**Additional file 1.** List of ≥ grade 3 non-irAE excluded for the definition of DLT

Any ≥ grade 3 non-irAE will be DLTs, except for the following conditions:

- Grade 3 fatigue lasting ≤ 7 days
- Grade 3 endocrine disorder (thyroid, pituitary, and/or adrenal insufficiency) that is managed with or without systemic corticosteroid therapy and/or hormone replacement therapy and the subject is asymptomatic
- Grade 3 inflammatory reaction attributed to a local antitumor response (e.g., inflammatory reaction at sites of metastatic disease, lymph nodes, etc.)
- Concurrent vitiligo or alopecia of any AE grade
- Grade 3 injection-related reaction (first occurrence and in the absence of steroid prophylaxis) that resolves within 6 hours with appropriate clinical management
- Grade 3 or 4 neutropenia that is not associated with fever or systemic infection that improves by at least 1 grade within 3 days. Grade 3 or grade 4 febrile neutropenia will be a DLT regardless of duration or reversibility
- Grade 3 or 4 lymphopenia
- Grade 3 thrombocytopenia that is not associated with clinically significant bleeding that requires medical intervention, and improves by at least 1 grade within 3 days
- Isolated grade 3 electrolyte abnormalities that are not associated with clinical signs or symptoms and are reversed with appropriate maximal medical intervention within 3 days
